# A consortium of detoxifying bacteria mitigates the aflatoxin B1 toxicosis on performance, health, and blood constituents of laying hens

**DOI:** 10.1016/j.psj.2023.102601

**Published:** 2023-02-16

**Authors:** Mohammad Amir Karimi Torshizi, Asghar Sedaghat

**Affiliations:** ∗ Department of Poultry Science, College of Agriculture, Tarbiat Modares University, Tehran 14115336, Iran; † Department of Poultry Science, University of Georgia, Athens, GA 30602, USA

**Keywords:** aflatoxin, laying hen, detoxification, performance, detoxifying bacteria

## Abstract

Detoxification approaches are evolving from physical to biological to eliminate the toxins altogether. The current study was conducted to compare the impact of 2 newly developed toxin deactivators, Magnotox-alphaA (**MTA**) and Magnotox-alphaB (**MTB**) with a commercially available toxin binder, Mycofix Plus^MTV INSIDE^ (**MF**) in alleviating the pernicious effects of aflatoxin B1 (**AFB1**) in laying hens. The treatments were: 1) negative control (**NC**; without AFB1), 2) positive control (**PC**; contaminated with 500 ppb AFB1), 3) MF (PC + 2 kg MF/ton feed), 4) MTA (PC + 2 kg MTA/ton feed), and 5) MTB (PC + 2 kg MTB/ton feed). Detoxifying bacteria revealed a substantial reduction of different toxins in vitro, in which 98.8, 94.5, and 73.3% degradation rates were achieved, respectively, for zearalenone (**ZEN**), patulin, and AFB1 in the first 1 h of exposure. The PC group had a sharp decline in egg production (**EP**; 68.83%) while MTB showed the superior EP (95.74%) followed by NC (90.66%), MF (86.57%), and MTA (82.08%; *P* ≤ 0.05). Egg weight (**EW**) was also observed to be inferior in PC group (53.80 g; *P* ≤ 0.05). Egg mass (**EM**) was higher in MTB (57.55 g) and NC (54.33 g) groups while PC produced the lowest (39.64 g; *P* ≤ 0.05). MTB and NC groups also demonstrated the best FCR, 1.62 and 1.68, respectively, and PC manifested the poorest FCR (1.98) with higher ADFI (*P* ≤ 0.05). MTB also produced a superior moisture content (**MC**; 82.11%) with inferior DM (17.89%) in ileum content (*P* ≤ 0.05). The greatest liver fat content was found in MF group (48.19%) and MTA yielded the superior serum β-carotene and Vit A. MDA level in yolk samples was influenced by treatments, rendering the highest level in PC group (*P* ≤ 0.05). Ileum microbiota and blood characteristics were also affected by treatments. In general, MTB proves to be a toxin-deactivator candidate with comparable results to that of commercially available toxin-binders.

## INTRODUCTION

Mycotoxins, metabolites of fungi, are ubiquitous in animal feed and cereal crops. Aflatoxins (**AFs**), as one prominent category of mycotoxins, are secondary metabolites of *Aspergillus flavus*, producing both aflatoxin B1 (**AFB1**) and aflatoxin B2 (**AFB2**), and *Aspergillus parasiticus*, furthermore producing aflatoxin G1 (**AFG1**) and aflatoxin G2 (**AFG2**). They are widely regarded to be severely immunotoxic, nephrotoxic, and hepatotoxic (Yang et al., 2020), among which, AFB1 is well-known for its carcinogenic and teratogenic mutation effects and its high degree of toxicity (Guan et al., 2021). To that end, WHO categorized it as a Class I carcinogen in 1993 (Cupid et al., 2004).

AFs could induce inflammatory damage to hepatocytes in case of long-term consumption of contaminated food, and trigger cancer cells to proliferate through AF-DNA adducts causing liver cancer (Hathout and Aly, 2014). Furthermore, an extensive cytotoxicity to neuronal cells such as apoptosis, S-phase arrest, DNA damage and ROS accumulation has been reported to be the cause of inducing apoptosis of CASP3 and BAX by AFB1 (Huang et al., 2020). AFs can also abolish the metabolic pathways of a wide assortment of intestinal microbiota, thereby causing certain metabolic diseases owing to their effects on energy supply (Akinrinmade et al., 2016).

AFs are mostly present in spices, nuts, and grains. The high humidity and temperature are contributing factors to promptly contaminating such feed/food (Zuo et al., 2013). The toxicity of AFs is not only observed in feeding, but also inpoisoned animals fed contaminated feed, and their by-products, leading to further severe spread of contamination in the food chain (Cherkani-Hassani et al., 2020). A concerted effort has recently been made to discover secure and highly effective approaches to detoxify feed/food. Finding practices to detoxify food safely and efficiently has thus become a focus of the research.

Various detoxifying techniques, including physical, chemical, and biological methods have developed as the focus of theresearch in the past few decades, all of which with their owndrawbacks; but the advantages of biological methods seem to outweigh their disadvantages (Guan et al., 2021). For instance, in physical methods, as the most common methods, the toxins are reported not being entirely absorbed along with their limited detox status, applicability, and poor detoxification. On the other hand, chemical approaches are demonstrated to leave chemical residue and compromise the taste and appearance of the food (Conte et al., 2020). Neither of such techniques seems to be the best approach for detoxification. Biological detoxification, however, is regarded as having a high degree of specificity, detoxifying the entire specimens, and producing innocuous products, albeit the safety of newly shaped products for the body and harboring its microbial performance are still of concern for researchers (Adebo et al., 2017; Conte et al., 2020). Therefore, it is considered the most appropriate approach to biological detoxification and has become the focal point among scientists. Microorganisms, often beneficial intestinal bacteria, are considered the best choice owing to their myriad crucial functions, such as preventing intestinal pathogens, boosting immune function and ameliorating the oxidative stress caused by mycotoxins, and preventing the ROS and RNS production (Afshar et al., 2020).

Many microorganisms, mostly those with probiotic properties, have been used for exerting detoxification and appear to yield promising results (Guan et al., 2021). *Bifidobacterium* strains are among such effective detoxifiers whose efficacy against AFB1, OTA, and patulin have already been reported (Hathout and Aly, 2014; Guan et al., 2021). Mycofix, designed by BIOMIN for poultry and swine, is the encapsulated *Eubacterium* sp. strain BBSH 797 and developed into a commercial formulation which is demonstrated to enhance the activity of rumen flora in dairy cows by acting against DON and substantially counterbalance the pernicious effects of T-2 toxin in broilers (Hathout and Aly, 2014).

Hence, having such beneficial microorganisms at our disposal and given the unprecedented expansion in the poultry sector and inevitable extensive use of feedstuffs which in turn elevates detrimental effects of AFB1, it is of critical importance to thwart such adverse effects in the poultry sector. The objectives of this study, therefore, were 1) to assess the efficacy of 2 newly developed products and exploit microorganisms’ 2-fold function including their beneficial probiotic properties and AF detoxification, and 2) to compare their potency with a commercially available mycotoxin deactivator in laying hens.

## MATERIALS AND METHODS

### In Vitro Aflatoxin Production, Culture Media, and Mycotoxins

*Aspergillus parasiticus* PTCC-5286 was administered to rice to produce AFB1 through fermentation. The approach to produce AFB1 on rice was adopted from Shotwell et al. (1966). Briefly, Erlenmeyer flasks containing sterile substrate were used and inoculated with a liquid suspension of mold spores. Two milliliters of such suspension was composed of 10^7^ spores/mL. After allowing cultures to grow at 25°C for 7 d with no illumination, the Erlenmeyer flasks were autoclaved and desiccated at 40°C for 48 h via a forced-air oven. The fine powder was achieved by grinding the culture material, and the level of AFB1 was determined via HPLC procedure, and then supplemented to basal diets to provide 500 ppb AFB1.

To investigate the in vitro efficacy of detoxifying bacteria, a minimal medium (**MM**) was used as a culture medium for the enrichment and isolation of mycotoxin-degrading strains. Ingredients were obtained from Sigma-Aldrich (Oakville, Ontario, Canada) and Sinopharm Chemical Reagent Co., Ltd. (Shanghai, China). Toxin standards for deoxynivalenol (**DON**), zearalenone (**ZEN**), Patulin, Ochratoxin A (**OTA**), AFB1, aflatoxin B2 (**AFB2**), aflatoxin G1 (**AFG1**), and aflatoxin G2 (**AFG2**) were purchased from Romer Labs (Erber Campus, Getzersdorf, Austria). Initially, 500 ppb of each mycotoxin was added to the medium containing detoxifying bacteria (2.2 × 10^9^ CFU/mL or 10^8^ CFU/mL) and then the concentration of each mycotoxin was measured after 1, 8, and 24 h of incubation. The internal SOP method was adopted to measure the concentration and reduction of each toxin in the media.

### Birds, Housing, and Management

The study protocol, procedures, and handling of the birds were conducted to meet the requirements set forth by the Animal Care and Use Review Committee guidelines of Tarbiat Modares University, Tehran, Iran. A 12-wk feeding study was carried out with a total of eighty-four 32-wk-old Bovans white laying hens, provided by a local commercial flock with similar average BW (1,530 ± 50 g), in 7 experimental groups with 6 replicate cages (2 birds per cage). Birds were housed in wire cages (40 × 45 × 45 cm) equipped with nipple waterer. All birds were fed with a commercial diet (soy-corn-based diets) and water ad libitum and were weighed at the beginning and at the end of the trial. Chickens were maintained at the recommended comfort temperature under a lighting program of 16L:8D. The basal diet was formulated to meet the requirements of Nutrition Specifications Bovans White layers Manual (Hendrix‐ISA, 2015), which is provided in Table 1.

**Table 1:** Ingredients and composition of basal diet fed to laying hens during experimental period, from 32 to 44 wk of age.

Items (% unless stated otherwise)	Of diet
Corn	60.03
Soybean meal (44% CP)	27.80
Soy oil	1.00
Limestone	8.83
Monocalcium phosphate	1.05
Sodium chloride	0.31
DL-Methionine	0.12
Mineral supplement^1^	0.25
Vitamin supplement^2^	0.25
Calculated chemical analysis	
Metabolizable energy (kcal/kg)	2750
Crude protein	16.80
Methionine + cystine	0.67
Lysine	0.89
Methionine	0.40
Threonine	0.65
Calcium	3.60
Available phosphorus	0.33
Sodium	0.21
Chlorine	0.15
Dietary electrolyte balance (mEq/kg)	194

1 The mineral premix supplied the following per kilogram of diet: 74.4 mg of Mn (manganese dioxide); 64.7 mg of Zn (zinc oxide); 75 mg of Fe (ferrous sulfate); 6 mg of Cu (copper sulfate); 0.2 mg of Se (sodium selenite); 0.87 mg of I (calcium iodate).

2 The vitamin premix supplied the following per kilogram of diet: 8,800 IU of vitamin A; 25,000 IU of vitamin D3; 11 IU of vitamin E; 2.2 mg of vitamin K_3_ (as menadione); 0.01 mg of cyanocobalamin; 4 mg of riboflavin; 0.46 mg of folic acid; 34.65 mg of pantothenic acid; 7.84 mg of niacin; 0.15 mg of biotin; 1.48 mg of thiamine; 2.46 mg of pyridoxine; and 200 mg of choline chloride.

The birds in different groups were offered the same basal diets with various AFB1 concentrations and the type of toxin-binder which are described in Table 2. The toxin-binders administered were a commercially available mycotoxin deactivator (Mycofix Plus^MTV INSIDE^, **MF**) containing yeast *Trichosporon mycotoxinivorans* (60 × 10^8^ count cells/g) obtained from Biomin GmbH, Austria. Two other mycotoxin deactivators were Magnotox-alphaA (**MTA**) and Magnotox-alphaB (**MTB**), provided by a commercial producer (Vivan Co., Mashhad, Iran). MTB and MTA were toxin deactivators with and without detoxifying bacteria, respectively. The bacterial complex incorporated into MTB included *Bacillus subtilis* (AL009126), *Bacillus licheniformis* (CP000002), *Bacillus coagulans* (CP009709), and *Bifidobacterium bifidum* (ATCC15696) with the concentration of 10^12^, 10^11^, 10^9^, and 10^9^ cfu/kg, respectively. Therefore, the treatment groups, the inclusion rates of AFB1, and toxin deactivators are summarized in Table 2.

**Table 2:** The inclusion rates of AFB1 and different toxin deactivators for treatment groups of the experiment.

Treatment	AFB1 (ppb)	Toxin deactivator (g/kg)
NC	-	-
PC	500	-
MF	500	Mycofix Plus^MTV INSIDE^ (2)
MTA	500	Magnotox-alphaA (2)
MTB	500	Magnotox-alphaB (2)

### Egg Production and Physical Egg Quality Characteristics

Eggs were collected, counted, and weighed daily to calculate egg production (**EP**, %) and egg weight (**EW**, g/egg). Average daily feed intake (**ADFI**, g/hen/day), egg mass (**EM**, g/hen/day; EW × EP), and feed conversion ratio (**FCR**, g feed/g egg laid; ADFI/EM) were also calculated. Values are represented on a per-hen basis.

Two eggs from each replicate, for a total of 12 eggs per group, were collected for assessment of physical quality characteristics. All the measurements were performed 3 times during the entire experiment with an equal timespan and an average for the whole period of the experiment was reported. All eggs were analyzed a day after collection. First, each egg was weighed intact and eggshell thickness was measured from 3 points of the egg being averaged, the small end, the large end, and the middle of the egg via an ultrasonic device (Echometer, Model 1061; Robotmation, Tokyo, Japan). Next, the egg was broken by an eggshell force gauge (Model-II, Robotmation, Tokyo, Japan) to measure the shell breaking strength and then the egg grade, yolk color, Haugh unit (**HU**), and albumen height were determined by the use of an egg multitester (EMT-5200; Robotmation, Tokyo, Japan). The HU was determined by the following formula:

$$\text{HU}=100\;\text{Log}\left(h+7.57-1.7\;w^{0.37}\right)$$

where h is the albumen height (mm) and w is the egg weight (g) (Haugh, 1937).

The shells were meticulously rinsed and dried at room temperature for 72 h and then the weights (inner shell membrane included) were recorded employing a digital scale (0.001 g). The proportion of shell, yolk, and albumen was calculated and expressed as the percentage of the egg. To steer clear of any subjective influence, all the egg quality measurements were made by the same individual throughout the whole trial.

### Sample Analysis

Determining dry matter (**DM**) and moisture portions (**MC**) of ileum content was conducted according to the Association of Official Analytical Chemists (method 934.01; AOAC, 1990). In brief, after sacrificing the birds (*n* = 6/treatment), 3 g of the sample from each replicate was smashed, homogenized, and placed into a previously dried and weighed moisture can and dried at 105°C via a convection oven (Memmert UNE 400, Memmert GmbH, Germany) until obtaining the constant weights. The MC for each sample was calculated in terms of the percentage of weight loss. The ether extract of the liver samples was also determined as stated byAOAC, 1990; method 920.39) by means of an apparatus (Soxtec system HT 1046 Tecator, Sweden) in order to report the liver fat content.

After collecting the blood and serum samples at the end of the experiment from each bird/replicate (*n* = 6/treatment), the measurement of β-carotene and Vit A was performed according to Jun-ichi Suzuki and Norio Katoh (1990). The analysis for blood biochemical characteristics (albumin, cholesterol, glucose, triglycerides, total protein, uric acid, and globulin) was conducted spectrophotometrically using commercial analytical kits (Pars Azmun Co., Tehran, Iran) and a microplate reader (Stat Fax 3200; Awareness Technology Inc., Palm City, FL) as per manufacturers’ instructions. The albumin-to-globulin ratio was also calculated.

At the end of the experiment, following the slaughter of the birds (*n* = 6/treatment) and removal of the ileum section in an aseptic environment, the diluted content was inoculated in selective agar media, and the number of colonies for total aerobic bacteria, *E. coli*, lactic acid bacteria, and spore formers was determined. The assessment was performed according to Sedaghat and Karimi Torshizi (2017). The concentration of malondialdehyde (**MDA**) was also determined in samples of egg yolk, thigh, and breast meat with iron-induced lipid oxidation (Sedaghat et al., 2016).

### Statistical Analysis

The data were fitted as a general linear model (**GLM**) procedure of SAS software version 9.4 (SAS Institute, 2014) to generate the least square means in response to the experimental diets with treatment means deemed significantly different at *P* ≤ 0.05. Each cage was considered an experimental unit. One-way ANOVA was performed in a completely randomized design adopting Duncan's multiple range test to compare the means and determine the significant difference, according to the following statistical model:

$$Y_{\text{ij}}=\mu +T_{i}+e_{\text{ij}}$$

where Y_ij_ is the dependent variable, μ is the overall mean, T_i_ is the fixed treatment effect, and e_ij_ is the residual.

## RESULTS

### In Vitro Study of the Culture Efficacy

**Figure 1:**
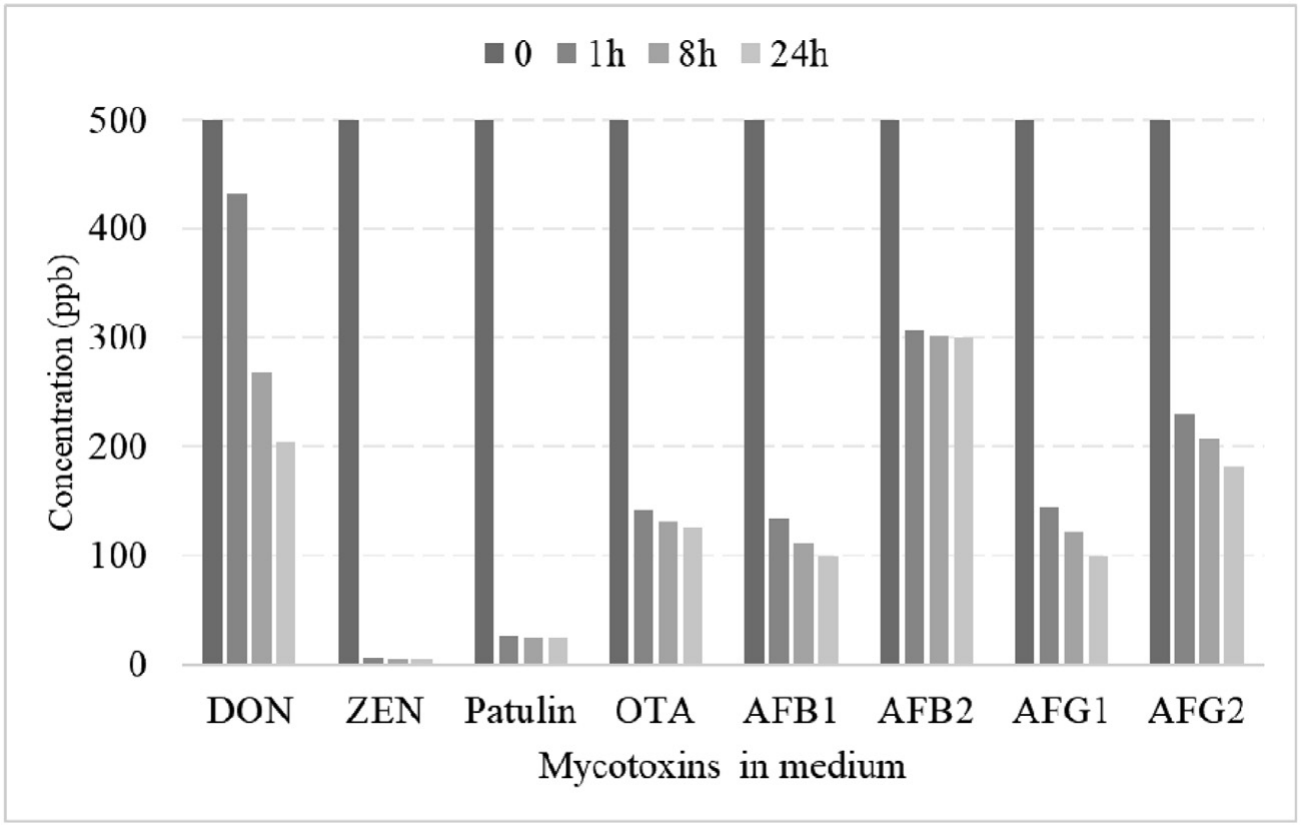
Degrading efficiency of the enriched culture. The concentrations of different mycotoxins in presence of detoxifying bacteria after elapsing 0, 1, 8, and 24 h of inoculation. The toxins DON, ZEN, OTA, AFB1, AFB2, AFG1, and AFG2 stand for deoxynivalenol, zearalenone, ochratoxin A, aflatoxin B1, aflatoxin B2, aflatoxin G1, aflatoxin G2, respectively. The initial concentration of each mycotoxin was 500 ppb with 2.2 × 10^9^ detoxifying bacteria in the medium.

The results of in vitro study on the efficacy of detoxifying bacteria on various mycotoxins, which are summarized in Figure 1, reveal that the complex of chosen bacteria was especially effective against ZEN and patulin in the first 1 h; 98.8 and 94.5% reduction in mycotoxins, respectively. This degradation rate after elapsing 24 h was, respectively, 58.9, 74.7, 80.1, 40, 79.9, and 63.7% for DON, OTA, AFB1, AFB2, AFG1, and AFG2.

### Performance

The outcomes of performance parameters demonstrated the lightest EW in the PC group (53.80 g; *P* < 0.05) while other treatments showed the heaviest EW, 60.18, 60.01, 59.38, and 57.73 g, respectively, for MTB, NC, MF, and MTA (*P* < 0.05; Table 3). The lowest ADFI was also observed in the PC group (77.67 g/d; *P* < 0.05) compared to others in which the highest ADFI was observed, 93.39, 91.24, 91.11, and 88.59 in MTB, MTA, NC, and MF, respectively (*P* < 0.05). The EP rate was observed to be the highest in the MTB group (95.74%, *P* < 0.05) along with NC and MF groups (90.66 and 86.57%; *P* > 0.05) whereas MTA and especially PC showed a significant drop in their EP (82.08 and 68.83%, respectively; *P* < 0.05). The changes in EM were also similar to EP, with the inferior EM in the PC group (39.64 g; *P* < 0.05) and the superior in the MTB group (57.55 g; *P* < 0.05). The EM for NC, MF, and MTA were 54.33, 51.36, and 49.02 g, respectively. For the FCR, however, the smallest numbers were observed in MF, NC, and MTB (1.72, 1.68, and 1.62, respectively; *P* < 0.05), and the largest ones were seen in PC and MTA groups (1.98 and 1.86, respectively). The ultimate BW of the PC group was significantly deteriorated (*P* < 0.05) whereas other treatment groups did not differ significantly in their ultimate BW (*P* > 0.05; data not shown for brevity).

**Table 3:** Laying performances of 32-wk-old hens among treatments of various toxin deactivators in tandem with AFB1 throughout a 12-wk feeding experiment.^1^

Treatment^2^	Egg weight (g)	ADFI (g/d)	Egg production (%)	Egg mass (g)	FCR (g/g)
NC	60.01^a^	91.11^a^	90.66^ab^	54.33^ab^	1.68^b^
PC	53.80^b^	77.67^b^	68.83^c^	39.64^c^	1.98^a^
MF	59.38^a^	88.59^a^	86.57^ab^	51.36^b^	1.72^b^
MTA	57.73^ab^	91.24^a^	82.08^b^	49.02^b^	1.86^a^
MTB	60.18^a^	93.39^a^	95.74^a^	57.55^a^	1.62^b^
SEM	0.83	1.66	2.52	1.58	0.03
*P* value	0.06	0.01	0.0009	<0.0001	0.0002

1 Data are means of 6 replications, with 2 hens per replicate.

2 Treatments NC, PC, MF, MTA, and MTB stand for negative control (neither AFB1 nor toxin deactivator); positive control (500 ppb AFB1 and no toxin deactivator); MF (500 ppb AFB1 with 2 g/kg Mycofix Plus^MTV INSIDE^); MTA (500 ppb AFB1 with 2 g/kg Magnotox-alphaA); MTB (500 ppb AFB1 with 2 g/kg Magnotox-alphaB), respectively.

a–c Values within a column with no common superscripts differ significantly (*P* < 0.05).

### Egg Characteristics, Liver Fat Content, and Vit A

Table 4 demonstrates the egg characteristics of the laying hens, in which shell strength was observed to be greater for NC, MF, and MTB, 4.39, 4.38, and 4.37 kg (*P* < 0.05), respectively, with the weakest shell being in PC and MTA (3.84 and 3.94 kg, respectively; *P* < 0.05). Albumen height was significantly superior for MTB (7.23 mm; *P* < 0.05) and inferior in the PC group (5.26 mm; *P* < 0.05). HU was also inferior in the PC group (67.9; *P* < 0.05) compared to other treatment groups. A significant increase in yolk color intensity was observed in the PC group (4.50; *P* < 0.05) and the lightest color was monitored in the MTB group (3.08; *P* < 0.05). There were no significant differences in yolk color densityamong NC, MF, and MTA groups (3.33, 3.42, and 3.42, respectively; *P* > 0.05). Shell weight was also different significantly among treatment groups (*P* < 0.05) from the heaviest in MF and MTB groups (6.23 and 6.09 g, respectively) to the lightest in MTA and NC groups (5.69 and 5.55 g, respectively). The largest proportion of albumen was observed in PC and MTB groups (62.53 and 62.79%, respectively; *P* < 0.05) and not significantly different from that of MTA and NC (61.18 and 61.81%, respectively; *P* > 0.05) and the smallest proportion of albumen was produced by MF group (60.07%; *P* < 0.05). The highest shell percentage of the egg belonged to the MF group (10.81; *P* < 0.05) with no significant differences among the rest of the treatment groups (*P* > 0.05). There were no significant differences in shell thickness, yolk weight, albumen weight, and yolk percentage (*P* > 0.05). The inferior egg grade was observed in the PC group (3.00, *P* < 0.05) whereas other treatments demonstrated significantly superior egg grades (3.92, 3.58, 3.83, and 3.88, respectively, in NC, MF, MTA, and MTB).

**Table 4:** Egg characteristics^1^ of 32-wk-old laying hens among treatments of various toxin deactivators in tandem with AFB1 throughout a 12-wk feeding experiment.^2^

Treatment^3^	ST (mm)	SS (kg)	AH (mm)	HU	YC	YW (g)	SW (g)	AW (g)	AP (%)	YP (%)	SP (%)	EG
NC	0.30	4.39^a^	7.04^ab^	83.75^a^	3.33^bc^	17.62	5.55^c^	37.59	61.81^ab^	29.03	9.16^b^	3.92^a^
PC	0.31	3.84^b^	5.26^d^	67.90^c^	4.50^a^	16.88	5.87^bc^	38.05	62.53^a^	27.79	9.68^b^	3.00^b^
MF	0.31	4.38^a^	6.18^c^	76.42^b^	3.42^b^	16.78	6.23^a^	34.76	60.07^b^	29.12	10.81^a^	3.58^a^
MTA	0.33	3.94^b^	6.75^b^	81.88^a^	3.42^b^	17.50	5.69^c^	36.55	61.18^ab^	29.30	9.52^b^	3.83^a^
MTB	0.32	4.37^a^	7.23^a^	83.95^a^	3.08^c^	17.20	6.09^ab^	39.37	62.79^a^	27.49	9.72^b^	3.88^a^
SEM	0.004	0.08	0.17	1.52	0.12	0.17	0.07	0.66	0.36	0.29	0.16	0.09
*P* value	0.55	0.01	<0.0001	<0.0001	<0.0001	0.46	0.003	0.24	0.10	0.15	0.01	0.0001

1 The terms ST, SS, AH, HU, YC, YW, SW, AW, AP, YP, SP, EG are, respectively, shell thickness, shell strength, albumen height, Haugh unit, yolk color, yolk weight, shell weight, albumen weight, albumen percentage, yolk percentage, shell percentage, and egg grade.

2 Data are means of 6 replications, with 2 hens per replicate.

3 Treatments NC, PC, MF, MTA, and MTB stand for negative control (neither AFB1 nor toxin deactivator); positive control (500 ppb AFB1 and no toxin deactivator); MF (500 ppb AFB1 with 2 g/kg Mycofix Plus^MTV INSIDE^); MTA (500 ppb AFB1 with 2 g/kg Magnotox-alphaA); MTB (500 ppb AFB1 with 2 g/kg Magnotox-alphaB), respectively.

a–c Values within a column with no common superscripts differ significantly (*P* < 0.05).

**Figure 2:**
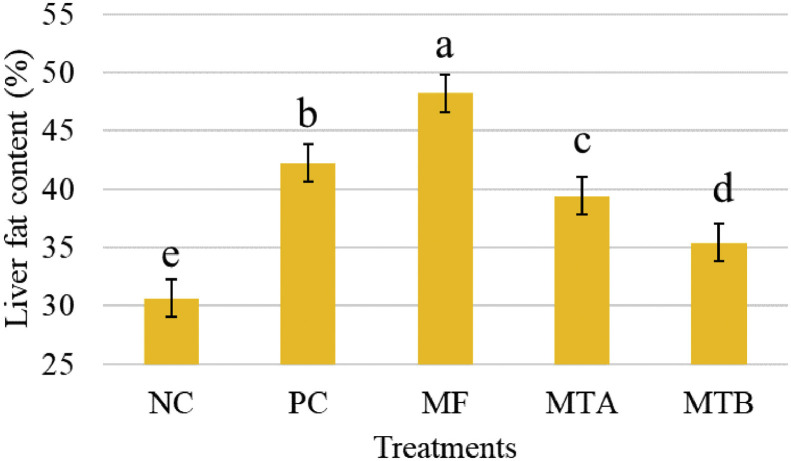
Liver fat content of 32-wk-old laying hens feeding various toxin deactivators in tandem with AFB1 for 12 wk. Treatments NC, PC, MF, MTA, and MTB stand for negative control (neither AFB1 nor toxin deactivator); positive control (500 ppb AFB1 and no toxin deactivator); MF (500 ppb AFB1 with 2 g/kg Mycofix Plus^MTV INSIDE^); MTA (500 ppb AFB1 with 2 g/kg Magnotox-alphaA); MTB (500 ppb AFB1 with 2 g/kg Magnotox-alphaB), respectively

There was a significant increase of liver fat content in the MF group (48.19%) followed by PC (42.21%), MTA (39.38%), and MTB (35.39%) while the lowest amount was found in NC (30.64%) group (*P* < 0.05; Figure 2).

### Ileum Content DM and Microbiota

**Figure 3:**
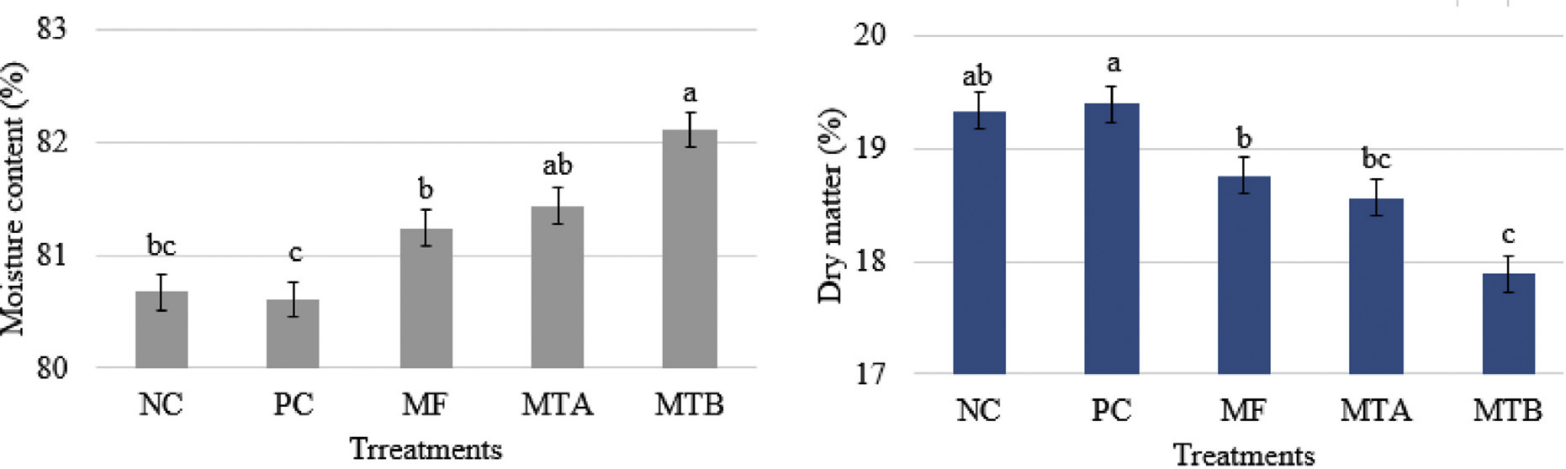
Moisture content and dry matter of the ileum content in 32-wk-old laying hens feeding various toxin deactivators in tandem with AFB1 for 12 wk. Treatments NC, PC, MF, MTA, and MTB stand for negative control (neither AFB1 nor toxin deactivator); positive control (500 ppb AFB1 and no toxin deactivator); MF (500 ppb AFB1 with 2 g/kg Mycofix Plus^MTV INSIDE^); MTA (500 ppb AFB1 with 2 g/kg Magnotox-alphaA); MTB (500 ppb AFB1 with 2 g/kg Magnotox-alphaB), respectively.

The results revealed that MTB produced a significantly superior MC (82.11%; *P* < 0.05) and the lowest DM (17.89%; *P* < 0.05) in ileum content when compared with other groups (Figure 3). On the other hand, PC and NC groups yielded a significantly low rate of MC (80.61 and 80.67%, respectively; *P* < 0.05) and the highest percentage of DM (19.39 and 19.33%, respectively; *P* < 0.05) among the treatments.

The results of the effects of a 12-wk feeding trial of toxin deactivators on the ileum microbiota population in 32-wk-old laying hens are provided in Table 5. The ileum microbiology assessment among treatments demonstrated a significant decline in total aerobic bacteria in the MF group (8.38 log_10_ CFU/g; *P* < 0.05) while the greatest population of total aerobic bacteria was found in the NC group (8.97 log_10_ CFU/g; *P* < 0.05). The greatest colony number of lactic acid bacteria was also observed in the NC group (8.30 log_10_ CFU/g; *P* < 0.05) followed by the PC group (8.26 log_10_ CFU/g; *P* < 0.05) whereas MF and MTB yielded the smallest populations of lactic acid bacteria (8.00 and 8.04 log_10_ CFU/g, respectively; *P* < 0.05) and *E. coli* bacteria (4.35 and 4.48 log_10_ CFU/g, respectively; *P* < 0.05). The group MTA demonstrated a significant rise in *E. coli* when compared with birds of other groups (5.84 log_10_ CFU/g; *P* < 0.05). Furthermore, the MF group was observed to possess an appreciable rise in spore former population (5.35 log_10_ CFU/g; *P* < 0.05) whereas NC and PC groups revealed the smallest populations of spore formers (4.41 and 4.57 log_10_ CFU/g; *P* < 0.05). In general, spore former bacteria were seen to be higher in treatments receiving toxin deactivators.

**Table 5:** Ileum microbiota in 32-wk-old laying hens among treatments of various toxin deactivators in tandem with AFB1 at the end of a 12-wk feeding experiment.^1^

Treatment^2^	Total aerobic bacteria	Lactic acid bacteria	*E. coli*	Spore formers
NC	8.97^a^	8.30^a^	5.18^b^	4.41^c^
PC	8.63^c^	8.26^ab^	5.17^b^	4.57^c^
MF	8.38^d^	8.00^c^	4.35^c^	5.35^a^
MTA	8.73^b^	8.19^b^	5.84^a^	5.05^b^
MTB	8.70^b^	8.04^c^	4.48^c^	5.09^b^
SEM	0.04	0.03	0.14	0.08
*P* value	<0.0001	<0.0001	0.0001	<0.0001

1 Data are means of 6 replications, with 2 hens per replicate. Colonies were enumerated and expressed as log_10_ CFU/g.

2 Treatments NC, PC, MF, MTA, and MTB stand for negative control (neither AFB1 nor toxin deactivator); positive control (500 ppb AFB1 and no toxin deactivator); MF (500 ppb AFB1 with 2 g/kg Mycofix Plus^MTV INSIDE^); MTA (500 ppb AFB1 with 2 g/kg Magnotox-alphaA); MTB (500 ppb AFB1 with 2 g/kg Magnotox-alphaB), respectively.

a–d Values within a column with no common superscripts differ significantly (*P* < 0.05).

### Serum β-Carotene and Vit A

**Figure 4:**
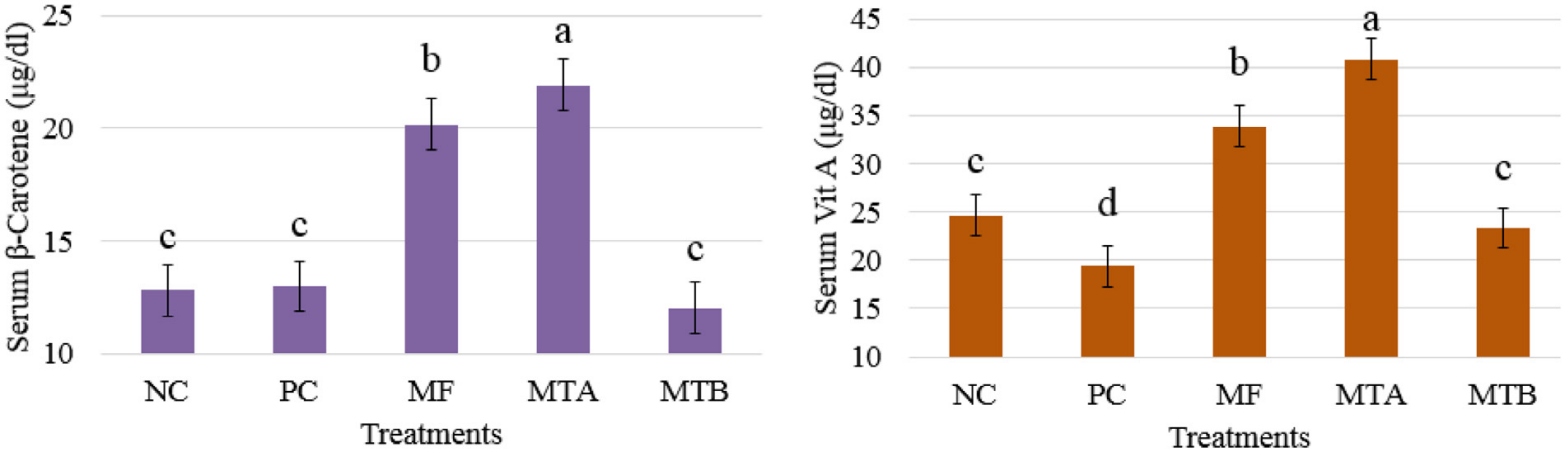
Serum β-carotene and Vit A of 32-wk-old laying hens feeding various toxin deactivators in tandem with AFB1 for 12 wk. Treatments NC, PC, MF, MTA, and MTB stand for negative control (neither AFB1 nor toxin deactivator); positive control (500 ppb AFB1 and no toxin deactivator); MF (500 ppb AFB1 with 2 g/kg Mycofix Plus^MTV INSIDE^); MTA (500 ppb AFB1 with 2 g/kg Magnotox-alphaA); MTB (500 ppb AFB1 with 2 g/kg Magnotox-alphaB), respectively.

Figure 4 shows that groups MF and MTA produced the highest concentrations of serum β-carotene (20.16 and 21.90 μg/dL, respectively; *P* < 0.05) while NC, PC, and MTB yielded the lowest levels of serum β-carotene (12.79, 12.98, and 12.02 μg/dL, respectively) compared to others (*P* < 0.05). The measurement of serum Vit A also revealed similar results in which the PC group demonstrated an inferior level (19.39 μg/dL; *P* < 0.05) whereas MTA and MF groups revealed superior levels of serum Vit A (40.81 and 33.83 μg/dL, respectively; *P* < 0.05).

### Yolk and Meat MDA Concentration

**Figure 5:**
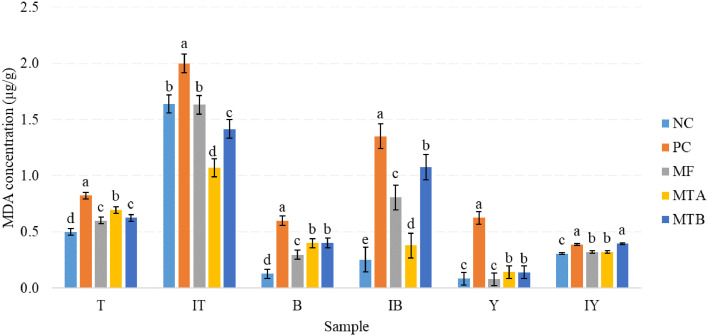
MDA concentrations of breast and thigh tissue and yolk samples of 32-wk-old laying hens feeding various toxin deactivators in tandem with AFB1 after 12 wk of experiment. Treatments NC, PC, MF, MTA, and MTB stand for negative control (neither AFB1 nor toxin deactivator); positive control (500 ppb AFB1 and no toxin deactivator); MF (500 ppb AFB1 with 2 g/kg Mycofix Plus^MTV INSIDE^); MTA (500 ppb AFB1 with 2 g/kg Magnotox-alphaA); MTB (500 ppb AFB1 with 2 g/kg Magnotox-alphaB), respectively. Samples T, IT, B, IB, Y, and IY stand for thigh, induced thigh, breast, induced breast, yolk, and induced yolk, respectively.

The results of MDA concentration in egg yolk, thigh, and breast meat samples, with or without iron-induced, revealed the highest and a significant level of MDA in the PC group in all samples (*P* < 0.05; Figure 5).

### Blood Biochemistry

The effects of supplementing various toxin deactivators in the presence of AFB1 in the feed of laying hens for a 12-wk feeding experiment on blood biochemistry parameters are summarized in Table 6. The results demonstrated a significant rise in albumin of the PC group (4.60 g/dL; *P* < 0.05) followed by the MTB group (4.17 g/dL; *P* < 0.05) and the lowest levels were observed in NC, MF, and MTA (4.04, 4.07, and 4.17 g/dL, respectively; *P* < 0.05). There were no significant differences in serum cholesterol and total protein among treatment groups (*P* > 0.05). Serum glucose concentration, however, significantly dropped in the PC group (137.49 mg/dL; *P* < 0.05) while maintaining higher in the rest of the groups (221.52, 242.50, 236.83, and 210.06 mg/dL for NC, MF, MTA, and MTB, respectively; *P* < 0.05). The concentration of triglycerides significantly decreased in PC birds (383.47 mg/dL; *P* < 0.05) while the other treatment groups exhibited higher amounts (820.45, 1,099.53, 1,018.89, and 778.54 mg/dL, respectively, for NC, MF, MTA, and MTB; *P* < 0.05). The highest concentrations of uric acid were observed in MTA, MF, and NC groups (7.12, 6.82, and 6.17 mg/dL, respectively; *P* < 0.05) while the PC group produced an appreciable lower level of uric acid (5.33 mg/dL; *P* < 0.05) which was not significantly different from MTB group (5.90 mg/dL; *P* > 0.05). The amount of globulin was revealed to be superior in the MTA group (3.08 g/dL; *P* < 0.05) with no significant differences with MTB, MF and NC groups (2.64, 2.34, and 2.29 g/dL, respectively; *P* > 0.05) while inferior globulin level was observed in PC group (2.09 g/dL; *P* < 0.05). The ratio of albumin to globulin tended to be the highest in the PC group (2.57; *P* < 0.05) and the lowest in the MTA group (1.34; *P* < 0.05) with other treatments scattered between these 2 treatments with no significant differences (1.83, 1.78, and 1.66 for NC, MF and MTB, respectively; *P* > 0.05).

**Table 6:** Blood characteristics of 32-wk-old laying hens among treatments of various toxin deactivators in tandem with AFB1 at the end of a 12-wk feeding experiment.^1^

Treatment^2^	Albumin (g/dL)	Cholesterol (mg/dL)	Glucose (mg/dL)	Triglycerides (mg/dL)	Total protein (g/dL)	Uric acid (mg/dL)	Globulin (g/dL)	Albumin: globulin ratio
NC	4.04^b^	166.25	221.52^a^	820.45^ab^	6.33	6.17abc	2.29^ab^	1.83^ab^
PC	4.60^a^	167.67	137.49^b^	383.47^c^	6.68	5.33^c^	2.09^b^	2.57^a^
MF	4.07^b^	182.95	242.50^a^	1099.53^a^	6.41	6.82^ab^	2.34^ab^	1.78^ab^
MTA	3.81^b^	170.97	236.83^a^	1018.89^ab^	6.89	7.12^a^	3.08^a^	1.34^b^
MTB	4.17^ab^	153.98	210.06^a^	778.54^b^	6.81	5.90^bc^	2.64^ab^	1.66^ab^
SEM	0.09	6.74	9.67	60.29	0.10	0.20	0.13	0.15
*P* value	0.05	0.78	0.0006	0.0001	0.35	0.02	0.10	0.09

1 Data are means of 6 replications, with 2 hens per replicate.

2 Treatments NC, PC, MF, MTA, and MTB stand for negative control (neither AFB1 nor toxin deactivator); positive control (500 ppb AFB1 and no toxin deactivator); MF (500 ppb AFB1 with 2 g/kg Mycofix Plus^MTV INSIDE^); MTA (500 ppb AFB1 with 2 g/kg Magnotox-alphaA); MTB (500 ppb AFB1 with 2 g/kg Magnotox-alphaB), respectively.

a–c Values within a column with no common superscripts differ significantly (*P* < 0.05).

## DISCUSSION

Taking advantage of beneficial bacteria, otherwise known as probiotics, has long been a subtle approach for safe production in the poultry sector and has recently become even more prominent to come under scrutiny by scientists to uncover other potential aspects of these beneficial bacteria. In the current research, we have attempted to depict a comparable newly developed toxin deactivator that is comprised of a consortium of detoxifying bacteria and how it exerts its influence in laying hens.

Detoxification of AFs using probiotics often encompasses multiple properties; hence, a combination of multiple species can be employed to obtain the most effective detoxification (Guan et al., 2021). Among various categories of probiotics, the use of LAB has extensively been studied and their respective mechanisms have already been spelled out (Shetty and Jespersen, 2006; Rashidi et al., 2020) with papers abound, but the research on *Bacillus licheniformis, Bacillus coagulans*, and *Bifidobacterium bifidum* is scarce. For instance, a study in which *B. megaterium, B. subtilis*, and *B. laterosporus* were used against AFB1 in Japanese quails revealed a better immune response, less oxidative stress, and less ileal spore-forming bacteria along with increased LAB population in ileum content comparable to that of commercially available toxin-binders (Razmgah et al., 2020). In the present study, the in vitro reduction of ZEN and patulin by the aforementioned bacterial complex in the first 1 h was a prelude to its assessment in an in vivo setting. The such effective reduction might have been due to degradation, absorption through the bacterial cell wall, and/or bonding with their secondary metabolites produced in the first growth phase. This reduction was even more conspicuous by elapsing time.

### Performance

Biodegradation of toxins by probiotics in broiler chickens is reported to improve digestibility, immune function, and growth performance and reduce the tissue and excreta residues of AFB1 when fed diets containing LAB (*Lactobacillus plantarum, Lactobacillus acidophilus*, and *Enterococcus faecium*) (Liu et al., 2018). An inferior growth performance and glutathione activity as the result of AFB1 activity and amelioration of such adverse effects via LAB strains (*L. acidophilus, L. plantarum*, and *E. faecium*) through detoxifying metabolism of AFB1 to AFM1 along with promoting glutathione activity leading to augmenting DNA protection has also been demonstrated (Liu et al., 2017). These are congruent with our results in which inferior performance parameters such as EP, EM, EW, ADFI, and FCR were observed in the PC group while other treatments demonstrated an improvement in such measurements. In fact, the superb performance was manifested in the superior EP, EM, EW, and FCR of the MTB group which were similar to the NC group. Therefore, it seems that the microbial complex has profoundly mitigated the deleterious effects of AFB1, leading to significantly improved performance parameters. It is stated that microorganisms can ameliorate the effects of AFs through regulating related pathways and also combining with AFs to hamper toxic effects of AFs (Guan et al., 2021). In addition, since mycotoxins are found in mixed types (e.g., AFs and DON, etc.) the use of multiple beneficial microorganisms would concurrently detoxify multiple toxins (Guan et al., 2021).

Our results of ameliorating the effects of AFB1 on BW and higher EP, EM, and EW are also in agreement with the study in which AFB1-detoxifying agents (hydrated sodium calcium aluminosilicate (**HSCAS**), mannan oligosaccharides (**MOS**), and *Lactobacillus acidophilus* (**LA**)) significantly mitigated the deleterious effects of AFB1 on final BW and BW gain of laying hens and increased EP, EW, and EM (Sherif et al., 2018). Similar to our study, their result of FCR was also improved. The detrimental effects of AFs on EP and egg grade have already been demonstrated in laying hens (Dazuk et al., 2021) and Japanese quail (Manafi, 2018) along with a poor FCR. Manafi (2018) has also found a lighter EW by AFB1 and heavier EW in a group receiving a toxin-binder (NBM) along with AFB1. Probiotics are believed to alter microbiota populations and the pH of GIT in favor of augmenting the activity of intestinal enzymes and nutrients’ digestibility, leading to an increase in ADFI and altering metabolic profile (Sherif et al., 2018).

### Egg Characteristics

A study using feed additives as detoxifiers such as HSCAS, MOS, and LA along with AFB1 reflected an increase in yolk weight, shell percentage, shell thickness, and yolk color (Sherif et al., 2018) which are partially in agreement with our results in which no significant differences were observed in yolk weight and shell thickness but MF group produced a significant rise in shell percentage, and an increase in yolk weight was manifested in only PC treatment. These discrepancies might be owing to the concentration and the type of detoxifiers. They also reported no significant alteration in albumen weight which is in line with the present study, but the result of the HU was at odds with the current results in which a significant decrease was seen in the HU by the PC group. The inferior value of the HU caused by AFB1 was in line with the experiment in quails (Manafi, 2018). This reduced score in the HU is presumed to be due to the effects of AFs on egg formation and interference in the normal course of fat mobilization from the liver to the ovary (Manafi, 2018). A lower yolk percentage and a higher proportion of albumen have been reported in laying hens as a result of AFB1 consumption (Dazuk et al., 2021) which are not concurrent with the current experiment in which no significant differences were observed in yolk percentages and the higher percentages of albumen were observed in PC, NC, and MTB. This discrepancy might be due to the dosage of AFB1 (2,510 vs. 500 ppb) and temporal exposure to AFB1 (42 d vs. 12 wk). The current data on eggshell percentage are in agreement with a decline in eggshell by AFB1 in laying quails (Manafi, 2018) but the data on shell strength are in conflict with that in which AFB1 caused no significant difference despite numerical lower values, whereas we observed a decline in shell strength by AFB1 alone. No effect on shell thickness in the present study was also concurrent with the study of Manafi (2018) in laying quails. These results may seem incongruous probably because of the bird type (quail vs. laying hens), the length of offering AFB1 (5 wk vs. 12 wk), and a variety of other confounding factors involving the experiment. The same argument could be proposed for blood cholesterol, triglyceride, glucose, and total protein. In addition, the albumen height is also gleaned from different studies to be adversely affected via AFs as it appears in the current study with the lowest value in PC group. Furthermore, a linear correlation is reported between poor egg quality, which exhibited in PC group in the current trial, and consuming AF (Manafi, 2018). The yolk color variation, as shown in the current study, is postulated to be associated with AFB1 intervention with lipid metabolism, deposition, and absorption of carotenoid in yolk (Sherif et al., 2018). In general, a disruption by AFB1 in reproductive performance and physiological systems has already been reported by Bagherzadeh Kasmani et al. (2018).

### Liver Fat Content and Vit A

Liver is believed to be the target tissue of AFs and a rise in its fat content is asserted to be one of the most prevalent symptoms of aflatoxicosis (Bagherzadeh Kasmani et al., 2018). In the case of current study, the most severe and elevated content of liver fat was observed in MF and PC groups, respectively, while MTA, MTB, and NC, in order, revealed the lower levels of fat content, indicating that MTA and MTB could significantly cushion the deleterious effects of AFB1. When comparing MTA and MTB groups, the latter demonstrated even lower concentration of liver fat content. This might be due to the microbial complex used in MTB. In fact, it is postulated that some probiotics could bind with AFB1 in GIT and be expelled from the body through excretion, and therefore lessen the severity of the effects of AFs (Bagherzadeh Kasmani et al., 2018).

Vit A, which plays a vital role in inhibiting the lipid peroxidation, is believed to be influenced by AFs. AFB1 diminishes the absorption of vitamins such as Vit A, thereby deteriorating the antioxidant defense mechanism (Bagherzadeh Kasmani et al., 2018). The present study demonstrated that MF and MTA could significantly elevate serum concentration of Vit A and MTB could prove the efficacy equal to NC group.

### Ileum Microbiology

The reduction of *E. coli* population in ileum microbiota by virtue of MF and MTB could be attributed to the beneficial effects of these treatments, especially the effects regarding microorganisms’ deactivation mechanism. In this context, Bagherzadeh Kasmani et al. (2018) revealed that the population of *E. coli* in the ileum decreased due to probiotics, stipulating that probiotics would adhere to the mucosal surface of the intestine and curb the colonization of *E. coli* via competitive exclusion. An increase in *E. coli* is believed to be the result of AF effects, which in turn is owing to enervating intestinal mucosal immunity, leading to a poor immune function and aggregation of harmful bacteria in upper GIT and a higher excretion rate via feces (Manafi, 2018). Probiotics and a cocultivation have been purported to be effective because of 3 mechanisms which are discussed and reviewed extensively by Guan et al. (2021). They maintain that using probiotics complex would not only improve the degradation rate of AFs, but also creates a more impervious epithelial barrier to either mycotoxins or other pathogenic toxins (Guan et al., 2021).

Contrary to the present results of lactic acid bacteria, AFs are suggested to reduce the population of beneficial bacteria and cells through peroxidation and producing free radicals (Manafi, 2018).

### MDA Content of Yolk and Meat

An elevated MDA concentration as a result of AFs consumption and mitigating their adverse effects via probiotics are reported by Bagherzadeh Kasmani et al. (2018) which is congruent with the current experiment in which MF, MTA and MTB groups significantly lowered the level of MDA. The same trend was also observed when samples underwent an iron-induced procedure. The beneficial effects of probiotics *Bacillus* spp. against AFB1 have also revealed in a study with Japanese quails (Razmgah et al., 2020). The synthesis rate of ceruloplasmin, a protein made by liver which stores and carries copper from the liver into the bloodstream and other parts of the body, and transferrin, the protein in the blood which binds to iron and transports it throughout the body, in the liver could be decreased through the inhibitory effect of AFs on protein synthesis, thereby raising the levels of free copper. This may lead to a decline in defense system to combat lipid peroxidation (Bagherzadeh Kasmani et al., 2018). Augmenting lipoperoxidation and diminishing antioxidant capacity was also demonstrated in a study with laying hens in which birds were exposed to AFB1 for 42 d (Dazuk et al., 2021). Therefore, a better function of liver through depressing MDA level by MF, MTA and MTB would seem to be the conclusion drawn from the current experiment.

### Blood Biochemistry

The result of increased serum globulin by the treatments in the current study is in line with the study in laying hens offered AFB1 and supplementing HSCAS, MOS, and LA as detoxifiers (Sherif et al., 2018) while the results of serum albumin, total protein, cholesterol, and uric acid were different, in which a drop in albumin level and a rise in uric acid and no significant difference in total protein and cholesterol were observed. A reduction in serum glucose, which is shown by PC group in the current trial, might be associated with the nephrotoxic effects of AFs, indicating a damage to the liver cells (Manafi, 2018), which is mitigated by other treatments.

## CONCLUSIONS

Using microbial complex, particularly those with probiotic properties, is an environmentally friendly, specific, effective, safe, and cheap strategy to be exploited in feed/food and poultry industries. In the current research, we have demonstrated that formulating an effective microbial complex, incorporated in MTB, to ameliorate the pernicious effects of AFB1 could not only equal but sometimes surpass that of commercially available detoxifiers reflected in performance parameters, liver fat content, MDA concentration in various samples, ileum microbiota and serum biochemistry. Even though this work is far from perfect, more thorough research is needed to especially address the mechanisms involved in detoxification, degrading enzymes produced by microorganisms and their nontoxic metabolites, and discovering more novel consortium of detoxifying bacterial preparations to maximize their efficacy against AFs and cushion or preclude the detrimental effects of toxins.
